# Prevalence of Early Childhood Caries in Children of West Godavari District, Andhra Pradesh, South India: An Epidemiological Study

**DOI:** 10.5005/jp-journals-10005-1372

**Published:** 2016-09-27

**Authors:** Srikanth Koya, KS Ravichandra, Vasa A Arunkumar, Suzan Sahana, HM Pushpalatha

**Affiliations:** 1 Senior Lecturer, Department of Pedodontics, Drs. Sudha & Nageswara Rao Siddhartha Institute of Dental Sciences, Allapuram, Andhra Pradesh, India; 2 Professor and Head, Department of Pedodontics, Drs. Sudha & Nageswara Rao Siddhartha Institute of Dental Sciences, Allapuram, Andhra Pradesh, India; 3 Reader, Department of Pedodontics, St. Joseph Dental College Duggirala, Andhra Pradesh, India; 4 Reader, Department of Pedodontics, St. Joseph Dental College Duggirala, Andhra Pradesh, India; 5 Senior Lecturer, Department of Pedodontics, Al-Badar Rural Dental College and Hospital, Kalaburagi, Karnataka, India

**Keywords:** Dietary habits, Early childhood caries, Oral hygiene practices, Prevalence.

## Abstract

**Aim:**

To determine the prevalence of early childhood caries (ECC) and its risk factors in children of West Godavari district, Andhra Pradesh.

**Study design:**

A sample of 1,897 children between 24 and 71 months of age based on stratified cluster random sampling were clinically examined for dental caries using mouth mirror under day light. The parents/caregivers of each child were interviewed with a structured questionnaire. Data was analyzed using independent sample Student’s t-test and chi-square test.

**Results:**

Out of the total 1,897 children, 796 were affected with ECC showing an overall prevalence of 41.9%, with boys showing a higher prevalence rate of 44.8% compared to girls (39.9%). Statistically significant correlations were found between ECC and consumption of sugary snacks (p < 0.0001) and mouth rinsing habit (p < 0.001).

**Conclusion:**

A higher prevalence of ECC was observed and most of the teeth were not restored.

**How to cite this article:**

Koya S, Ravichandra KS, Arunkumar VA, Sahana S, Pushpalatha HM. Prevalence of Early Childhood Caries in Children of West Godavari District, Andhra Pradesh, South India: An Epidemiological Study. Int J Clin Pediatr Dent 2016;9(3):251-255.

## INTRODUCTION

Dental caries affects humans of all ages across the world and the complex multifactorial etiology associated with its initiation and progression makes it difficult to eradicate.^1,2^ Dental caries in young children has a unique pattern and poses entirely new challenges. It is the single-most common chronic childhood disease and is five times more frequent than asthma and seven times than hay fever. Infants with early childhood caries (ECC) may be underweight, suffer from iron deficiency, and grow at a slower pace because of their disinclination to eat due to associated pain.^3^

Since 1962, a variety of terms have been used to describe caries in young children, including the terms baby bottle tooth decay, nursing bottle syndrome, bottle mouth caries, nursing caries, rampant caries, nursing bottle mouth, milk bottle syndrome, breast milk tooth decay, maternally derived *Streptococcus mutans* disease (MDSMD) and faciolingual pattern of decay and about 106 factors have been associated with its etiology.^4^ To standardize the terminology and to better represent the multifactorial etiology, the term early childhood caries (ECC)^*^ has been recommended to describe any form of caries in infants and preschool children.

Epidemiological studies reveal considerable variation in the prevalence of caries in preschool children ranging from 3 to 85% with strong correlation to socioeconomic status and ethnicity.^5-10^ One of the recent studies from Scotland has reported decline in the prevalence of dental caries in 3-year-olds which can be attributed to the preventive program undertaken locally.^11^ This highlights the importance of prevalence studies and understanding of the local factors influencing ECC in designing a preventive program.

The rationale for conducting the present study was, very few prevalence studies have been reported in India on ECC and none so far in the southern state of Andhra Pradesh, India. The present study was undertaken in the West Godavari District of Andhra Pradesh, with the aim to study the prevalence of ECC and also to analyze the influence of factors, such as diet, oral hygiene measures, and social factors on the prevalence of ECC.

## MATERIALS AND METHODS

The present study was designed and conducted by the Department of Pedodontics and Preventive Dentistry, St. Joseph Dental College, Eluru, in an attempt to determine the prevalence of ECC in children aged between 24 and 71 months. A total of 2,052 schools are present out of which 1,728 are anganwadi (Government) and 324 are private schools. A pilot study was conducted to calculate the sample size and to assess the feasibility of the study. Sample size for the main study was calculated based on the prevalence’s of ECC in pilot study. A minimum sample size of 1,800 was required to have 80% power and 95% CI. One private and five anganwadi schools were selected to achieve proportionate representation by simple random method from 11 mandals, out of 46 mandals in the West Godavari district and every 3rd child were included in the study through systematic sampling. A total of 1,897 children, including 794 boys (42%) and 1,103 girls (58%) (Tables 1 and 2) were screened after obtaining written consent from parents/caregivers and approval from district educational officer (DEO) and project director (PD) of anganwadi schools. The dentition status of WHO oral health assessment form (1997) was used to record dental caries.^12^ The study was conducted between June and September 2012. Ethical approval was obtained from the Institutional Ethics Committee.

The children were examined on a chair under broad day light by using sterile dental mouth mirrors after drying the teeth with a cotton wool. All the children were examined by the same examiner and 10% of children were re-examined to minimize the intra-examiner variability.

A two-part questionnaire, modified according to the Indian conditions, was used in this study.^2^ Data on social factors was recorded by interviewing the parents/caregivers in the first part while information pertaining to the brushing habits, diet intake, and deft score of the child was included in the second part. The data was statistically analyzed to find the prevalence of ECC and to determine the correlation, if any, between ECC and brushing habits, dietary practices, and the social factors.

Statistical analysis was done with Statistical Package for the Social Sciences (SPSS) version 17 (Chicago Inc.). Student’s t-test was used for comparison between continuous variables, and chi-square test was used for categorical data.

## RESULTS

Out of the total 1,897 children (Table 1), 796 were affected with ECC showing an overall prevalence of 41.9%, with boys showing a higher prevalence rate of 44.8% compared to girls (39.9%). However, there was no statistically significant association between ECC and sex of the child (Table 2).

**Table 1:** **Table 1:** Gender distribution of study participants

*Gender*			*Participants*	
Male			794 (41.9%)	
Female			1,103 (58.1%)	
Total			1,897 (100%)	

**Table 2:** **Table 2:** Early childhood caries prevalence

*Gender*		*Number*		*No. with* * ECC*		*Total no.* * with ECC*		*Mean* *DMFT± (SD)*	
Boys		794 (42%)		356 (44.8%)		796 (41.9%)		1.51 ± 2.44	
Girls		1,103 (58%)		440 (39.9%)					
Total		1,897							

**Table 3:** **Table 3:** Relationship between ECC and diet

*Diet*		*Total* * number of* *children*		*No. of* * children* * having* * caries*		*% of* * children* * having* * caries*		*Mean* * DMFT ± (SD)* * of DEFT*	
Sugary		418		417		99.8		5.28 ± 2.39	
Nonsugary		1,479		379		25.6		0.44 ± 0.92	

The impact of diet on prevalence of ECC was evaluated based on the sweet score of snacks consumed by the children. The total sample was grouped into children consuming sugary diet (sweet score > 15) and nonsug-ary diet (sweet score < 15). The sugary diet group consisted of 418 (22%) children while the nonsugary diet group consisted of 1,479 (78%) children. In addition, 417 children of the cariogenic diet group (99.8%) and 379 (25.6%) of the noncariogenic diet group were affected with ECC, revealing significant positive correlation between ECC and consumption of the cariogenic diet (p < 0.0001) (Table 3).

Out of the total sample, 832 (43.9%) children had their mother/caregivers assistance during brushing while 1,065 (56.1%) children performed unaided brushing; 330 (39.7%) children with aided brushing and 466 (43.8%) of the unaided group had ECC, wherein no significant association was found between ECC and mode of tooth-brushing. Only 8.2% (156) children of the total sample rinsed mouth after snacking. Thus, 29 children (18.6%) of the mouth rinsing group and 767 (44.1%) of the non-rinsing group exhibited ECC. A statistically significant association was seen between ECC and the mouth rinsing habit after snacks (p < 0.001) (Tables 4 and 5).

Parental literacy patterns revealed 1,092 mothers/ caregivers (57.5%) and 938 fathers/caregivers (49.4%) were uneducated. The prevalence of ECC in children of maternal literacy was more (49.1%) compared to illiteracy (36.7%). In contrast, ECC was less in children of educated fathers (35.6%) compared to uneducated (48.5%). However, statistically, no significant association was found between ECC and literacy patterns. Out of the total sample, 970 children (51.1%) were enrolled in private schools while 927 (48.8%) were in government schools; 414 children (42.7%) of the private schools and 382 (41.2%) of the government schools had ECC. The prevalence of ECC was found to be slightly more in children going to private schools compared to the children going to the government schools. However, statistically significant association cannot be made between ECC and the type of school attended by the child (Tables 6 and 7).

**Table 4:** **Table 4:** Relationship between ECC and brushing

*Brushing*		*Total number* * of children*		*No. of* * children* * having* * carise*		*% of* * children* * having* * caries*		*Mean ± SD*	
Aided		832		330		39.7		1.38 ± 2.34	
Un-aided		1,065		466		43.8		1.61 ± 2.51	
Total		1,897		796		41.9		1.51 ± 2.44	

**Table 5:** **Table 5:** Relationship between ECC and mouth rinsing

*Mouth* * rinsing*		*Total number* * of children*		*No. of children* * having caries*		*% of children* * having caries*		*Mean ± (SD)* *of deft*	
No		1,741		767		44.1		1.58 ± 2.46	
Yes		156		29		18.6		0.76 ± 2.02	

**Table 6:** **Table 6:** Relationship between ECC and type of school attended

*Type of school*		*No. of* * children*		*No. of children* * with ECC*		*Percentage*	
Private		970		414		42.7	
Government		927		382		41.2	

**Table 7:** **Table 7:** Relationship between parent’s education and caries

*Parents education*		*Total* *number of* * children*		*No. of* * children* * having* * caries*		*% of* * children* * having* * caries*		*Mean ± (SD)* * of DEFT*	
Mother education									
Uneducated		1,092		401		36.7		1.57 ± 2.49	
Educated		805		395		49.1		1.45 ± 2.38	
Father education									
Uneducated		938		455		48.5		1.58 ± 2.56	
Educated		959		341		35.6		1.41 ± 2.26	
Total		1,897		796		41.9		1.51 ± 2.44	

## DISCUSSION

Dental caries patterns in the developed world are very well understood because of the regularity with which the prevalence studies are being conducted and the established diet habits. Also, some of the preventive programs undertaken at the community level started yielding results and declining trends in dental caries have been reported.^12^ Contrary to this, dental caries in the developing world has become a new threat and is on the rise because of the easy availability of refined sugars than earlier, compromised primary level of prevention, very few community-based programs to eradicate the disease and the lack of public knowledge about the disease. In addition to this, the dental caries patterns are least understood due to very few prevalence studies conducted, differing local factors in the western world, and rapid change in the dietary habits. This study was undertaken with a view to find the prevalence of dental caries in children and the local factors influencing it since the factors influencing dental caries in this area differ from other parts of India and no such study had been conducted earlier in this rural region.

School-going children between 24 and 71 months of age were chosen for this study because of their easy accessibility and to ensure uniformity in sampling. A pretested, validated questionnaire consisting of structured open-ended questions covering oral hygiene/dietary habits, oral health behaviors, and sociodemographic variables was preferred to interview the parents/ caregivers of the children instead of self-administered questionnaire to eliminate nonresponses, misconceptions, and errors.

Studies across the world have demonstrated varied caries prevalence in the preschool children ranging from 3% to 85%.^5-9,13^ The ECC prevalence in this study was 41.9%, which was lower than that of the Far East Asian countries but considerably higher than Nordic countries, such as Finland and Sweden.^5-9,13^ Also, the results correspond to other studies in India where an overall prevalence of 44% had been reported with a tendency toward lower ECC prevalence in urban areas than rural.^9,14^ Very few children with ECC had received restorative treatment. This can be attributed to the lack of public knowledge about primary prevention as well as preventive programs and less access to dental clinics as most of them are urban-centric. The increased prevalence of ECC in boys compared to girls may be associated to the gender bias favoring males regardless of the socioeconomic class which manifests itself in longer feeding of boys compared to girls in the Indian society.

The present study showed high correlation between ECC and the cariogenicity of diet, which was assessed by measuring the sweet score which is in line with several earlier studies.^12,15^ Children were given sweets frequently as a reward or on demand by mothers who constituted the majority of caregivers in this study, a predominantly culture-based practice. Also, the indiscriminate use of pediatric medicines and syrups which are mostly cariogenic may also be a contributing factor for ECC.^1^ This highlights the importance of parent counseling regarding the judicious use of sugary snacks and pediatric medicines for the well-being of general and oral health of the child.

In the present study, children who performed aided brushing had slightly lower prevalence of ECC and similar findings have been reported in other studies as well.^14^ This may be due to recall bias as well as social desirability response bias of the answers given by the parent/caregiver. Also, the duration and type of brushing technique/dentrifice which have a bearing on the results were not evaluated in this study. On the contrary, a significant reduction in ECC prevalence was observed in children who mouth rinsed after snacking, though the percentage of children following this habit was less. This is in accordance with earlier studies.^16^

Contrary to the studies showing inverse relationship between educational attainment of parents and ECC, no statistical association can be drawn between ECC and the literacy of parent/caregivers.^14^ On the other hand, children of educated mothers in this study were having slightly higher prevalence of ECC. This may be due to the cultural practice of frequent rewarding of the child in the form of sugary snacks that suggests a lack of awareness/ negligence about healthy oral hygiene practices, which needs to be addressed.

In previous studies, socioeconomic factors have been identified as predisposing factors in the development of both dental caries and periodontal disease.^17^ High income families in industrialized countries experience less caries than those of low income, a trend that appears to be quite opposite in the developing countries.^17,18^ The social class of the children is based on the assumption that the children attending the government schools belong to low-income group since the parents/ caregivers were reluctant to reveal their incomes. It was observed that the prevalence of ECC was slightly more in the children attending the private schools comprising of relatively high-income groups when compared to the children attending government schools with low-income groups, though statistical significance was not observed. This observation contradicts a majority of earlier studies found in the literature and is suggestive of the changing diet patterns of the newly emerging middle class of this region that needs to be addressed.^17,18^

Another trend observed, though not related to the study was higher rate of enrollment of girls into government schools than private schools, confirming the role of gender bias of Indian society where preferential treatment to the male child is given.

## CONCLUSION

An overall ECC prevalence of 41.9% between the ages of 24 and 71 months was observed in the children of West Godavari district, Andhra Pradesh, South India. Observations like very few restored teeth, consumption of sugary diet by the child which was encouraged predominantly by the mother, and lack of rinsing after snacking among majority of children were suggestive of the lack of awareness about healthy oral practices in this region. Also, the increased tendency of children of high-income families toward ECC was relevant to the changing diet habits of the newly emerging middle class. Some of the cultural issues like gender bias and active mother’s role in the child rearing practices were some of the local factors influencing ECC. The findings from this study indicate the urgency of creating a preventive program to restore the carious teeth as well as to motivate and educate the children/caregivers toward healthy oral hygiene practices.

However, further studies including the severity of the carious lesions in terms of age, detailed investigation of the oral hygiene practices, and socioeconomic factors encompassing a larger population have to be carried out to design a preventive program suitable for this area.
